# A Lanthanum-Tagged Chemotherapeutic Agent HA-Pt to Track the *In Vivo* Distribution of Hyaluronic Acid Complexes

**DOI:** 10.24947/2380-5552/1/1/102

**Published:** 2015-03-03

**Authors:** Ti Zhang, Qiuhong Yang, W.C. Forrest, Shuang Cai, Daniel Aires, M. Laird Forrest

**Affiliations:** 1 Department of Pharmaceutical Chemistry, The University of Kansas, Lawrence, KS, USA; 2 HylaPharm LLC, Lawrence, KS; 3 Department of Internal Medicine, The University of Kansas Medical Center, Kansas City, KS, USA

**Keywords:** Hyaluronic acid, Lanthanum, Biodistribution, Human head and neck squamous cell carcinoma

## Abstract

Hyaluronic acid drug conjugates can target anti-cancer drugs directly to tumor tissue for loco-regional treatment with enhanced bioavailability, local efficacy and reduced toxicity. In this study, the distribution and pharmacokinetics of hyaluronic acid carrier and a conjugated cisplatin anti-cancer drug were tracked by lanthanum (III) [La(III)] affinity tagging of the nanocarrier. The strong binding affinity of La(III) to HA enabled the simple preparation of a physiologically stable complex HA-Pt-La and straightforward simultaneous detection of HA-La and Pt in biological matrices using inductively coupled plasma-mass spectrometry (ICP-MS). Consequently, after subcutaneous injection of HA-Pt-La nanoparticles in human head and neck squamous cell carcinoma (HNSCC) tumor-bearing mice, the HA and Pt content were detected and quantified simultaneously in the plasma, primary tumor, liver and spleen.

## Introduction

The dissemination of cancer cells, referred to as cancer metastasis from the primary tumor to distant organs through the bloodstream and lymphatic vessels is the leading cause of cancer death [1]. Compared with blood vessels, lymphatic vessels and their draining lymph nodes (LNs) are more prominent in developing metastatic growth and spreading metastases of epithelial cancers. The detection of metastases in regional or sentinel lymph nodes (SLNs) indicates a worse prognosis with a decreased chance of survival compared to undisseminated disease. In fact, lymphatic vasculatures not only act as conduits to distant sites in the body for tissue-invading tumor cells, but also play a critical role in facilitating the dissemination of cancer cells [2]. Peritumoral and intratumoral lymphatic networks are short of drainage capability due to their disorganized microvessels similar to tumor blood vasculatures. However, the structural irregularity disrupts the normal flow of tumoral lymphatic vessels and increases the susceptibility to invasion by malignant cells.

In head and neck squamous cell carcinoma (HNSCC), lymphatic metastasis is a critical prognostic factor for patients’ survival, due to the preferential spread of malignant cells to the roughly 60-70 lymph nodes in the head and neck area. Lymphangiogenesis is found to occur in most clinical cases of HNSCC, wherein heterogeneously distributed intratumoral and peritumoral lymphatic vessels were identified [3]. The lymphagiogenic growth factors in HNSCC could be present throughout almost the entire tumor, with especially high expression of VEGF-C and D at the invasive front tumor region [4].

The glycosaminoglycan hyaluronic acid (HA) is an endogenous extracellular matrix mucopolysaccharide that plays a critical role in inflammatory responses, wound healing and neoplasia. HA is a principle ligand for CD44, which is overexpressed in many cancer cells [5]. HA also targets the lymphatic vessel endothelial hyaluronan receptor (LYVE-1) for clearance in the lymphatic system [6-8]. LYVE-1 is expressed specifically on the lymphatic endothelium and has been used as a molecular marker in studies of lymphatic trafficking and tumor-induced lymphangiogenesis [9]. LYVE-1 plays an important role in the uptake of HA by afferent lymphatic endothelial cells. After uptake by lymphatic vessels, HA is mobilized, turned over and catabolized within draining lymph nodes, before entering circulation for clearance by the liver [10]. Thus, HA has been used as a targeted nanocarrier for the delivery of anticancer agents to lymphatic metastases, such as lymphatically metastatic breast cancer and HNSCC. HA-Platinum (HA-Pt) nanoparticles have been shown to prolong lymphatic retention and improve tumor tissue deposition [10]. Moreover, the high water solubility of HA facilitated the subcutaneous (s.c.) administration of HA-Pt for loco-regional treatment, so that the bioavailability and efficacy of nanoconjugates were dramatically improved compared to intravenous cisplatin (CDDP) [10,11]. For example, mice bearing HNSCC xenografts had significantly increased intratumoral concentration of Pt post s.c injection of HA-Pt compared to the animals treated with i.v. CDDP [12]. It is probably due to the formation of a HA-Pt drug depot after the local injection, which released the drug sustainably and delivered the Pt into cancer cells via lymphatic vessels surrounding the tumor. However, the precise biological disposition of HA has not yet been fully elucidated.

The *in vivo* biodistribution of HA has been tracked and quantified using fluorescent imaging agents, such as near infrared (NIR) fluorescent dyes, quantum dots (QDots), and after isotopic labeling [13-16]. For example, a HA-OPots800 conjugate, synthesized via an adipic acid dihydrazide (ADH) linker, was orally administrated for real-time bioimaging to investigate the optimal molecular weights and extent of chemical modification of HA for an efficient drug delivery [13]. HA has also been labeled with radioactive isotopes including ^99m^Tc, ^3^H, ^111^In, ^125^I and ^11^C to track its biodistribution with relatively high detection sensitivity and specificity [17-21]. For instance, Melendez-Alafort *et al.* labeled a HA-paclitaxel conjugate with ^99m^Tc to evaluate its biodistribution through four different administration routes by measuring *ex vivo* gamma-ray activity in organs and conducting *in vivo* gamma ray image analysis [22]. However, due to fluorescent quenching of dyes in physiological environments, high toxicity of QDots and the safety concerns of isotope uses, there is a critical need to develop an effective and non-toxic approach to *in vivo* tracking HA.

In the current study, our goal is to look into the distribution pattern of HA nanoparticles and correlate it with the distribution pattern of Pt. Lanthanum chloride (LaCl_3_) has been used by Tohoku *et al.* to effectively extract HA from the defatted rabbit skin [23]. We built on this ground work and harnessed the strong binding affinity of the lanthanum(III) [La(III)] to HA and prepared a physiologically stable complex HA-Pt-La via non-covalently doping a trace amount of La(III) to the HA-Pt conjugates. The binding affinity of the La(III) to the HA-Pt conjugates was evaluated using an *in vitro* release test. In addition, after s.c. injection of HA-Pt-La nanoparticles in HNSCC tumor-bearing mice, the Pt(II) and La(III) content were simultaneously tracked and quantified in the plasma, primary tumor, liver and spleen using a highly sensitive and reliable inductively coupled plasma-mass spectrometry (ICP-MS) technique. The high specificity and sensitivity of the ICP-MS analysis enables the accurate determination of low-abundance Pt and La [below parts per trillium (ppt)] in the native biological samples.

## Method and Materials

### Materials

All chemicals were obtained from commercial suppliers and used without further purification unless otherwise noted. HA (35 kDa) was purchased from Lifecore Biomedical (Chaska, MN) as sodium hyaluronate (NaHA), which was produced by a microbial fermentation process. CDDP was obtained from AK Scientific (Union, CA). Lanthanum(III) nitrate hexahydrate (La(NO_3_)_3_·6H_2_O) (puriss. p.a., ≥99.0%) was purchased from Sigma-Aldrich Co (St. Louis, MO). All other chemicals and cell culture supplies were purchased from Sigma-Aldrich Co (St. Louis, MO) or Fisher Scientific (Pittsburgh, PA). Deionized distilled water (ddH_2_O) was used in syntheses, cell culture (sterilized by autoclaving) and animal experiments (sterilized by autoclaving). Human HNSCC cell line MDA-1986 was kindly provided by Dr. Jeffery Myers (The University of Texas, M.D. Anderson Cancer Center, Houston, TX).

### Synthesis of HA-Pt-La Conjugate

The HA-Pt conjugate was prepared as previously described [10]. Briefly, 100 mg of NaHA and 45 mg of CDDP were dissolved in a total of 20 mL of ddH_2_O and stirred in the dark for 96 h under argon at ambient temperature (ca. 25°C). At the end of the reaction period, the mixture was filtered through a 0.22-μm nylon membrane filter (Fisher Scientific; Pittsburgh, PA), followed by dialysis (MWCO 10,000 Da; Pierce, IL) against ddH_2_O for 48 h in the dark with four water exchanges. To synthesize the HA-Pt-La, 1.36 mg of La (NO_3_)_3_·6H_2_O (1.1 eq to polymer. 3.14 μmol) was added to the HA-Pt aqueous solution. The pH of the mixture was adjusted to pH 5.5 using 0.1-N NaOH, and the mixture was stirred overnight protected from light at ambient temperature (ca. 25°C). The unreacted La(III) was removed by dialysis against ddH_2_O for 48 h in the dark. The crude HA-Pt-La was concentrated under reduced pressure by rotary evaporation and then stored at 4°C in the dark. The substitution degrees of Pt and La were determined by ICP-MS analysis (Agilent Technologies 7500 i, Santa Clara, CA) using terbium as the internal standard and high purity argon (>99.996%) as the carrier gas.

### Characterization of HA-Pt-La Conjugate

The molecular weight and the polydispersity index (PDI) of HA-Pt-La were determined by Gel Permeation Chromatography (GPC) on a Shimadzu 2010CHT HPLC with a refractive index (RI) detector (Shimadzu RID-10A) and UV detector at 210 nm. GPC was performed with a Shodex OHpak SB-804 HQ column (Showa Denko America, Inc., New York, NY) at 40°C using 5-mM ammonium acetate buffer (pH 5.0) as the mobile phase at a flow rate of 0.8 mL/min. A calibration curve was generated with HA polymers with molecular weights ranging from 6,400 – 132,000 g/mol.

To observe the morphology of the HA-Pt-La nano-conjugate, a drop of 10-mg/mL HA-Pt-La solution in ddH_2_O was placed on a lacey carbon coated copper grid (200 mesh, TED PELLA, Redding, CA). Transmission electron microscope (TEM) images were recorded using a FEI Tecnai F20 XT Field Emission TEM (FEI, Hillsboro, Oregon) at an accelerating voltage of 200 ekV.

### *In vitro* Release Profile of Pt and La from the HA-Pt-La Conjugate

The *in vitro* release rates of the hydrate form of CDDP (*cis*-[Pt(NH_3_)_2_(OH_2_)_2_]^2+^) and La(III) from the HA-Pt-La conjugate were determined using a dialysis method. Typically, 1 mL of HA-Pt-La solution was added into the dialysis tubing (MWCO 10,000 Da) and then placed in a 2.0-L phosphate-buffered saline (PBS) (pH 7.4) solution at 37°C with stirring at a speed of 300 rpm. The bath volume was replaced every 12 h to maintain a sink condition. A 50-μL aliquot was withdrawn from the dialysis tubing at predetermined time points. The Pt and La concentrations in each sample were determined by ICP-MS analysis.

### Cellular Toxicity of HA-Pt-La Conjugate in the HNSCC Cancer Cells

Human HNSCC MDA-1986 cells were cultured in modified Eagle’s medium alpha (supplemented with 10% fetal bovine serum and 1% L-glutamine) and seeded into 96-well plates at a density of 5000 cells/well. After incubation at 37°C in a humidified, 5% CO_2_ incubator for 24 h, cells were treated with HA-Pt-La at concentrations from 0.0065 to 195 μM (on cisplatin basis). Seventy-two hours post-treatment, a resazurin blue solution was added into each well with a final concentration of 5 μM. After 4 h incubation, the fluorescence signal (ex/em, 560/590 nm) in each well was measured using a fluorophotometer (SpectraMax Gemini, Molecular Device, Sunnyvale, CA). Trichloroacetic acid and PBS were used as positive and negative controls, respectively. The IC_50_ value was determined as the midpoint between the positive and negative controls. Each experiment was repeated in triplicate.

### Induction of Human Xenografts of HNSCC Tumor

All experimental procedures were approved by the University of Kansas Institutional Animal Care and Use Committee (IACUC). Female NU/NU mice (20-25 g, Charles River Laboratories; Wilmington, MA) were anesthetized with 2% isoflurane in oxygen, and 50 μL of a suspension of MDA-1986 cells (2 × 10^7^ cells/mL) was injected subcutaneously into the oral submucosa using a 30-ga needle. Tumor growth was monitored twice per week by bi-dimensional measurement with a digital caliper, and the tumor volume was calculated using the equation:

$$\text{Tumor volume}({\mathrm{mm}}^{3})=0.52\times {\left(\text{width}\right)}^{2}\times (\text{length}).$$

### Evaluation of Pharmacokinetics and Tissue Distribution of HA-Pt-La in HNSCC Tumor Bearing Mice

HA-Pt-La was mixed with HA-Pt before administration with a final overall loading degree of 9.7% for Pt and 0.09% for La on a weight basis. When head and neck tumors grew to a size range of ca. 100 to 150 mm^3^, animals were randomly divided to two groups, including a non-treated group (N = 3) and drug-treated group (N = 3 per timepoint). In the drug-treated group, HA-Pt-La was administered s.c. peritumorally with a single dose of 1mg/kg on a cisplatin basis. The animals were euthanized at 0.25, 1, 6, 24 and 48 h post injection.

Whole blood was drawn and centrifuged at 2,000×g for 5 min to collect the plasma. Tumors, livers and spleens were also harvested, washed with PBS and stored at −80°C until analysis. To determine the Pt and La levels in the tissues and plasma samples, approximately 20 mg of freeze-dried tumor and spleen tissues, 100 mg of freeze-dried liver tissues or 100 μL of plasma were digested with 0.5 mL of aqua regia at 130°C for 2 h. Subsequently, the digested samples were diluted using 1% HNO_3_ and analyzed by the ICP-MS.

## Results

### Syntheses and Characterization of HA-Pt-La Conjugate

The HA-Pt-La was prepared using a two-step synthesis. Pt(II) was first conjugated to the HA with a conjugation efficiency of 27% and a loading degree of 9.96 wt. % through forming a liable ester linkages with the polycarboxyl groups of the HA polymer. However, the loading degree of Pt(II) decreased from 9.96 wt. % to 7.40 wt.% in the final HA-Pt-La conjugate, probably due to the slow Pt release from the HA backbone during the 5-day process of production and purification [24]. In the meantime, the release of Pt liberated a number of carboxylate groups on the HA, which in turn facilitated the strong binding of La(III) to the oxygen atoms of the carboxylate groups [25]. The conjugation degree of La(III) was determined to be 0.37 wt.%.

**Figure 1:**
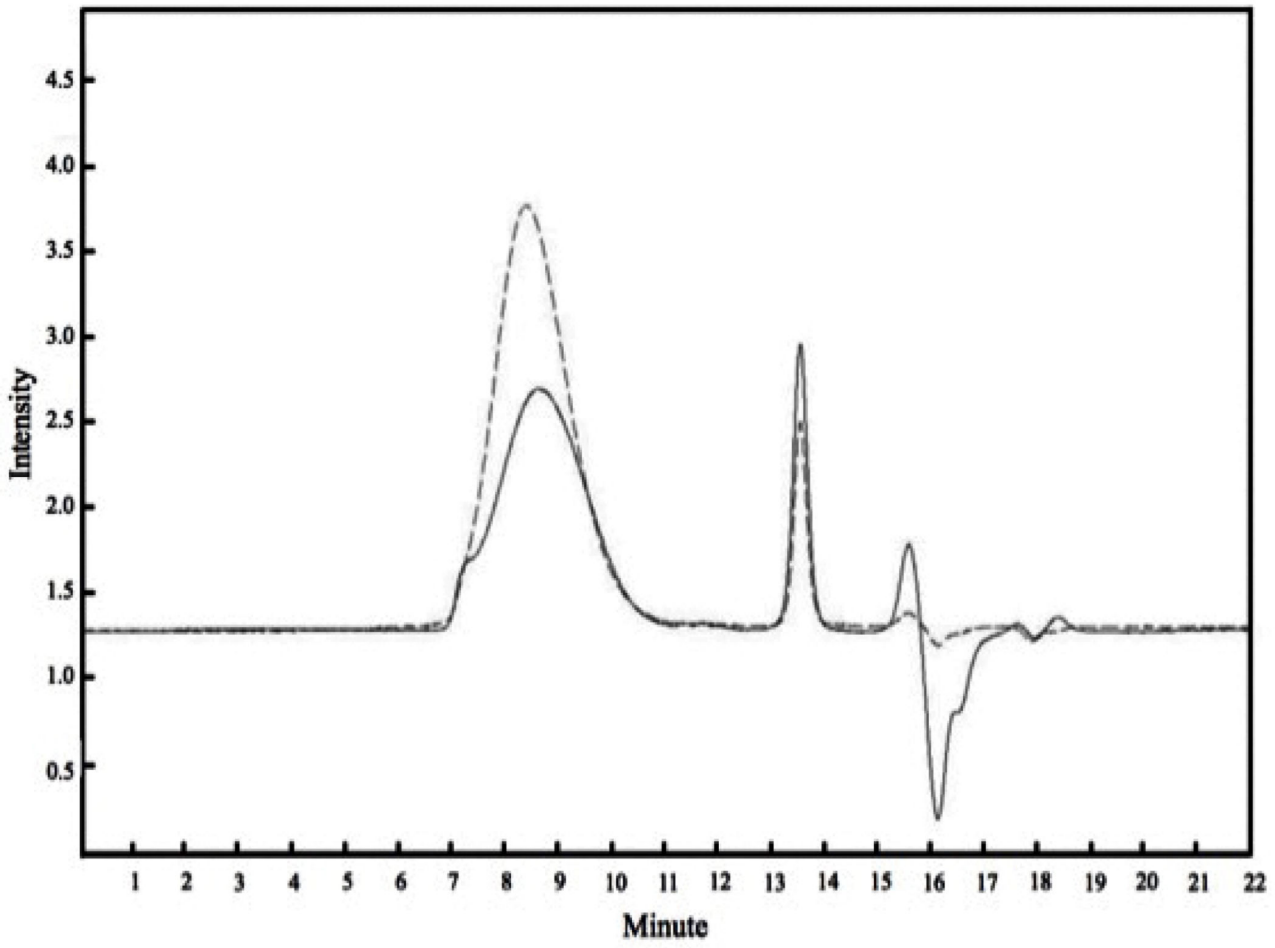
Chromatograms of NaHA (dashed) and HA-Pt-La conjugates (solid) generated by a GPC with a RI detector.

**Figure 2:**
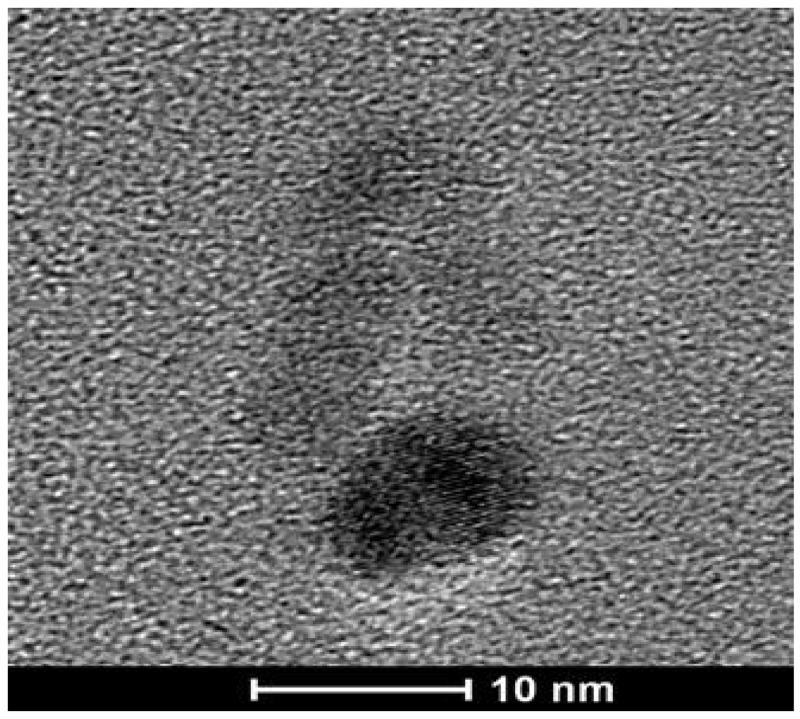
TEM image of HA-Pt-La conjugates. The scale bar is 10 nm.

Compared with the native HA, the obtained HA-Pt-La sample had a comparable molecular weight as confirmed by GPC (Figure 1). Specifically, its weight-average (M_w_) and number-average molecular weights (M_n_) were 35 kDa and 24 kDa, respectively, resulting in a PDI of 1.44 (Table 1). Both NaHA and HA-Pt-La had broad elution range and thus high PDI values. This was probably due to the viscous drag, a non-size exclusion effect found on viscous polymers [26]. TEM images of the HA-Pt-La conjugate showed a spherical shape with a size of approximately 10 nm (Figure 2), which was within the optimal range for lymphatic uptake and nodal retention, 10 to 80 nm in diameter [27].

### *In vitro* Release Profile of Pt and La from the HA-Pt-La Conjugate

**Figure 3:**
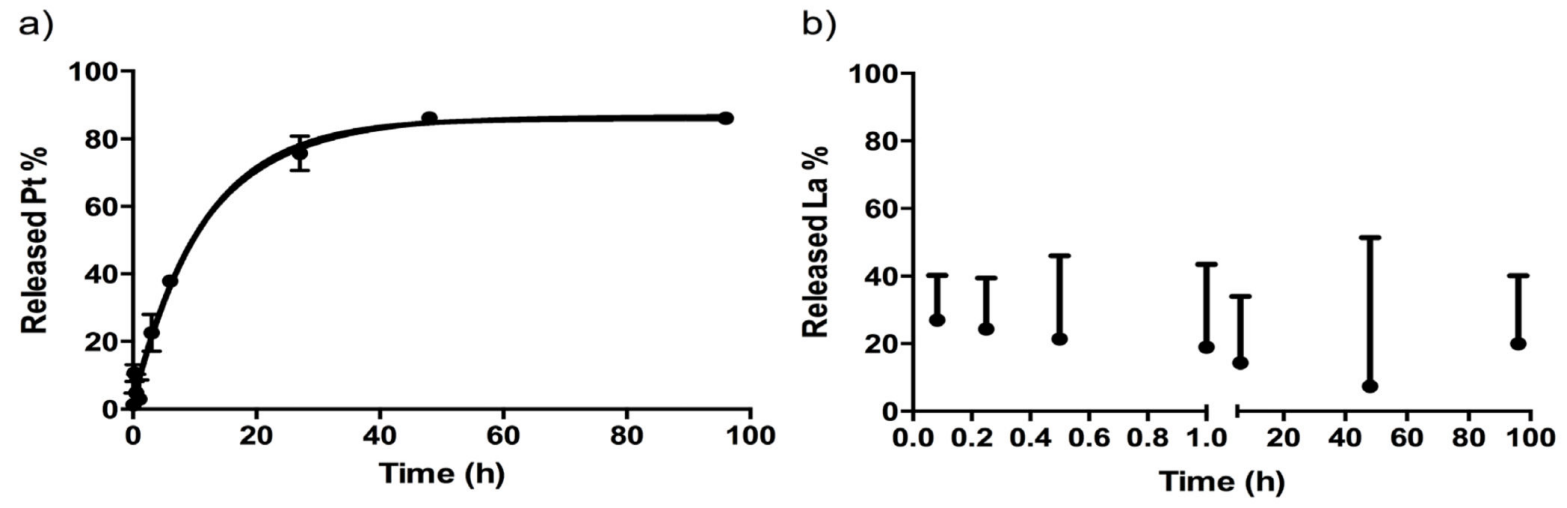
*In vitro* release profiles of a) Pt(II) and b) La(III) from HA-Pt-La conjugate in PBS (37°C).

The *in vitro* release profiles of Pt(II) (7.40 wt.%) and La(III) (0.37 wt.%) in the PBS medium are shown in Figure 3. Pt(II) release from the HA-Pt-La complex could be fit to a pseudo-first-order release model with a half-life of approximately 10 h and a rate constant of 0.068 h^−1^ (Figure 3a). The anions in the PBS, including phosphate and chloride, rapidly displaced the carboxyl groups that bind to the Pt, leading to the relatively shorter release half-life of Pt upon hydrolysis. In comparison, La(III) exhibited an initial burst release of approximately 20% within first 30 min, and no further La(III) release was detected in the following 4 days (Figure 3b). The stable HA-La(III) binding suggests that the La-labeled HA-Pt conjugate, HA-Pt-La, could be used as a detection probe to monitor the *in vivo* distribution of the HA by measuring the La levels in the plasma, tumor and organ tissues.

### Cellular Toxicity of HA-Pt-La Conjugate

**Figure 4:**
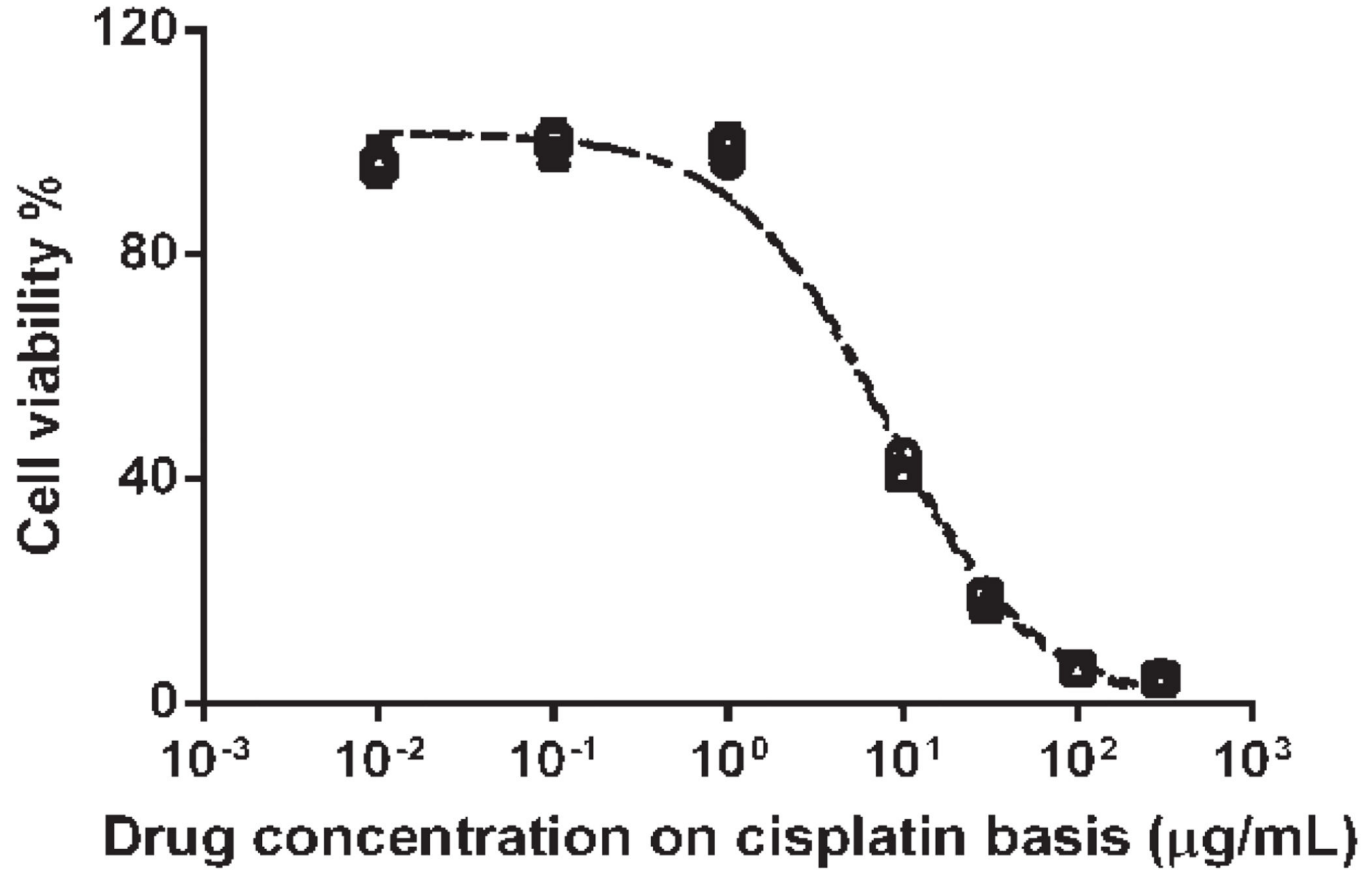
Inhibition of HNSCC MDA-1986 cell growth by the HA-Pt-La conjugate after 72 h incubation.

The cytotoxicity of HA-Pt-La in the highly metastatic HNSCC cell line MDA-1986 was determined as the reduction in cell proliferation (Figure 4). The HA-Pt-La conjugates have an *in vitro* IC_50_ value of ca. 7.81 ± 0.24 μM on a cisplatin basis, which was not significantly different from the reported IC_50_ values of the free CDDP (6.6 μM) or HA-Pt (6.0 μM) [10].

### Pharmacokinetics and Tissue Distribution of HA-Pt-La Conjugate

**Figure 5:**
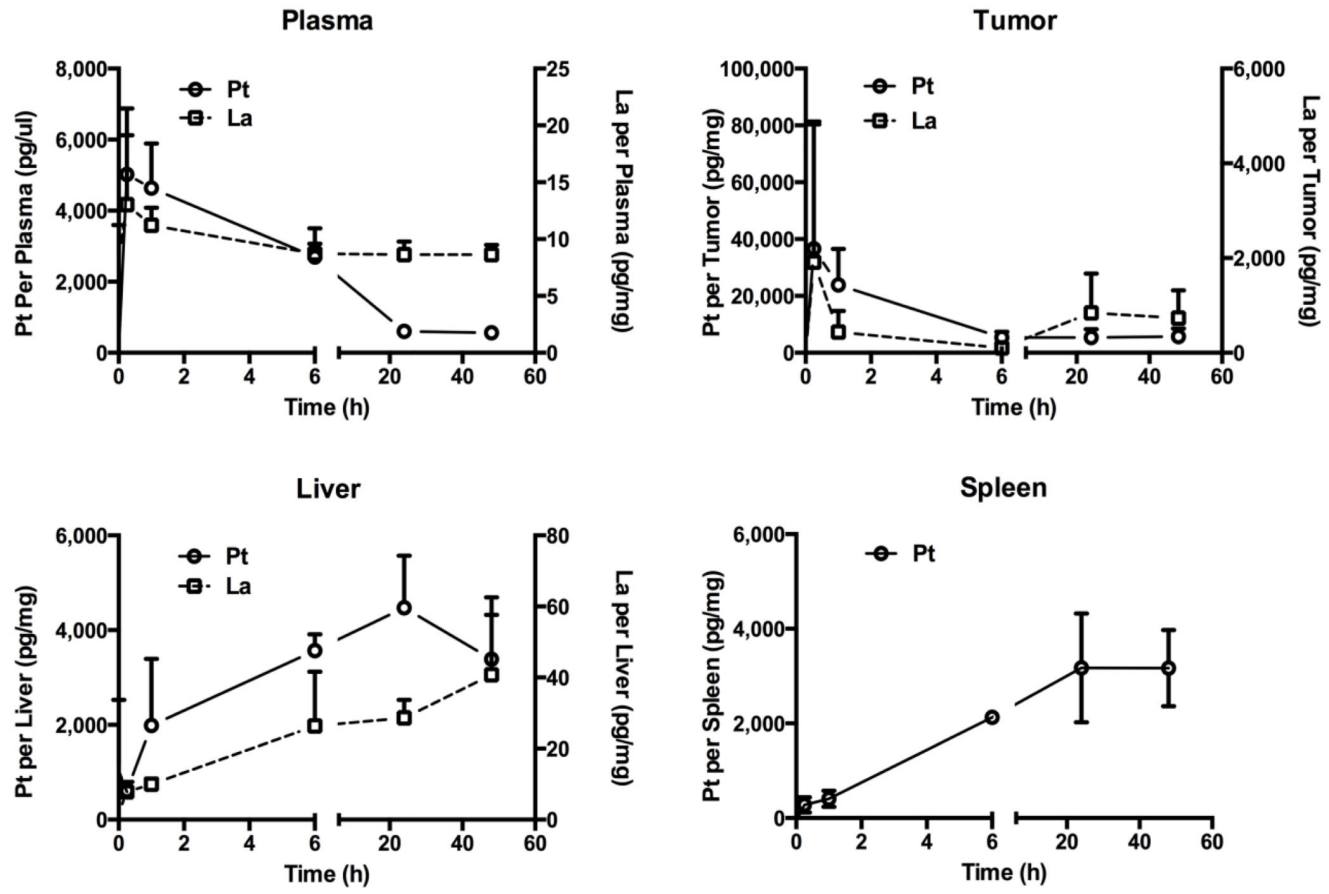
Tissue and plasma concentrations of Pt and La after s.c. injection of the mixture of HA-Pt and HA-Pt-La conjugates (1.0 mg/kg on cisplatin basis) into the tumor area. Of note, La data was not presented in the figure of spleen samples as its count was below the quantification limit.

A sub-therapeutic level of 1 mg/kg (on cisplatin basis) [10] was used to investigate the *in vivo* pharmacokinetics and tissue distribution of the HA-Pt-La conjugate. The La contents in tumor, liver and spleen samples were measured at different time points to capture the bio-distribution pattern of HA in living mice (Figure 5). In the tumor and plasma samples, peak concentrations of La and Pt following the s.c. administration both occurred at 15 min, due to their initial burst-release from the disintegrated HA in the tumor region. As expected, the released Pt was rapidly cleared and its concentration in the tumor and plasma samples decreased to near-baseline levels within 24 h post-injection. In comparison, the concentrations of La in the plasma decreased in a much slower rate, which indicated that HA had a higher plasma residence time and slower clearance in the blood circulation. Moreover, within the first hour post-injection, only a small amount of Pt and La accumulated in the liver and spleen, which correlated with their high levels detected in tumor and plasma. As liver and spleen are the major organs for macromolecular accumulation, disintegration and clearance by the hepatobiliary system [16], the released Pt and the La-labeled HA gradually accumulated in the liver and spleen within 6 h post-injection and almost reached the plateau at 24 h.

The pharmacokinetics data were modeled by SAAM II software using a three compartmental model. The three compartments include an injection site compartment, a central plasma compartment, and a third body tissue compartment. Several transfer rate constants were applied to the model to establish a relationship between compartments, for example, a k(1,3) between the injection site and the plasma compartment, k(1,2) and k(2,1) between the plasma and the tissue compartments, and a loss constant of k(0,1) from the plasma compartment representing the elimination of the drug (Figure 7). The pharmacokinetic parameters of Pt in mice, clearance, plasma area under the curve (AUC), half life (t_1/2_) and steady-state volume of distribution (V_ss_), are summarized in Table 2.

## Discussion

The lanthanides (Ln) are a series of metallic chemical elements, which include 15 elements from La through Lu. The most attractive property of lanthanide for biomedical application is the capability of its trivalent ions to bind with oxygen-donor ligands or less stable nitrogen-donor ligands to form a coordination complex, Ln(III), which is kinetically and thermodynamically stable in the blood. The unique fluorescence properties of Ln ion complexes [Ln(III) chelates], such as large Stokes shifts and long emission lifetimes, make them well-suited for biomedical imaging with the minimum self-fluorescence interference from biological fluids and tissues. In addition, due to its high magnetic moment and long electron relaxation time, the Ga(III) ion has been employed in the noninvasive radiological examination technique-nuclear magnetic resonance imaging (NMRI) [25]. Among the chelates for Ln(III), macrocyclic polyaminocarboxylic ligands, such as DOTA (1,4,7,10-Tetrakis(carboxymethyl)-1,4,7,10-tetraazacyclododecane), are capable of forming the most stable complexes due to the size of their internal cavities, their conformation, as well as their rigidity [28]. Recently, a Ln-chelating carbohydrate conjugate based on a phenylene diamino tetraacetic chelating unit was explored to characterize the carbohydrate formation and its interactions with proteins in solutions, and it has demonstrated the successful formation of stable Ln(III) ion complex with four carboxylic groups [29]. HA-La complex, on the other hand, can been synthesized by reacting La(III) ion to the oxygen atoms of the carboxylic groups on the D-glucuronic acid units. Although there was no further binding to nitrogen atoms of the macrocycle, HA-La complex has shown an excellent stability of up to 4 days in the physiological pH and ionic strength in our study. This result could be explained by the nature of HA structure, where the negatively charged carboxylate groups and the spatial restrictions around the glycosidic bonds coil up into a stiff structure that is called random coil in biological environments [30]. This arrangement creates a spatial allowance for more carboxylate groups binding to La(III) and provides a barrier to prevent the diffusion of hydrolyzed La(III) from the “HA cage”. Moreover, forming the HA-Pt-La complex did not induce the intermolecular cross-linking of HA polymers, which was verified by the negligible difference in molecular weights and PDIs between the native HA and HA-Pt-La complex.

HA exists in the cartilage scaffolding, the synovial fluids of joints and the extracellular matrix. HA-specific receptors enable the targeted delivery of anti-cancer drugs via conjugating to HA nanocarriers, thus reducing the systemic toxicity. These receptors include CD44, the receptor for HA-mediated cell motility (RHAMM), and HA receptor for endocytosis (HARE) to mediate the HA uptake in the liver [31]. In addition, HA-drug bioconjugates give rise to the enhanced drug solubility and stability, improved localization and controlled release. Previously it has been shown that after i.v. administration to rodents or rabbits, HA enters the blood stream, from where it was taken up and removed by the endothelial cells. HA is degraded mainly in the liver and is also concentrated in the spleen and lymph nodes [32]. However, due to the rapid sequestration of injected compound in the liver, the i.v. administration route was not considered as a suitable approach for the systemic treatment of tumors and metastases spreading out in the body. In our previous studies, HA-Pt conjugates were peritumorally injected, and the tumoral uptake of Pt was substantially improved as evidenced by the increased AUC when compared with the i.v. cisplatin route [24].

We proposed that upon the loco-regional administration, the HA-Pt conjugate might be retained in the close anatomical district from where Pt was sustainably released from the HA depot, and then delivered into cancer cells via then lymphatic vessels and eventually form a Pt-DNA adduct inside the nucleus [12]. As shown in Figure 5, Pt was cleared from primary tumor region with a comparable rate as HA, which is in line with our proposed tumoral uptake mechanism. Once entering the systemic circulation, HA level in the plasma, however, decreased much slower than Pt with a stable level reached 6 h post-injection. Nonspecific uptake of HA nanoparticles is mainly by the reticuloendothelial system (RES), which is particularly represented in the liver and spleen. In addition, HARE receptors that interact with the HA backbone are primarily present in the hepatic tissues. Moreover, subcutaneously injected HA is naturally taken up by the lymphatics, followed by the transport to the systemic circulation, and finally enzymatically degraded in the liver. Indeed, HA exhibited gradual accumulation in the liver.

**Figure 6:**
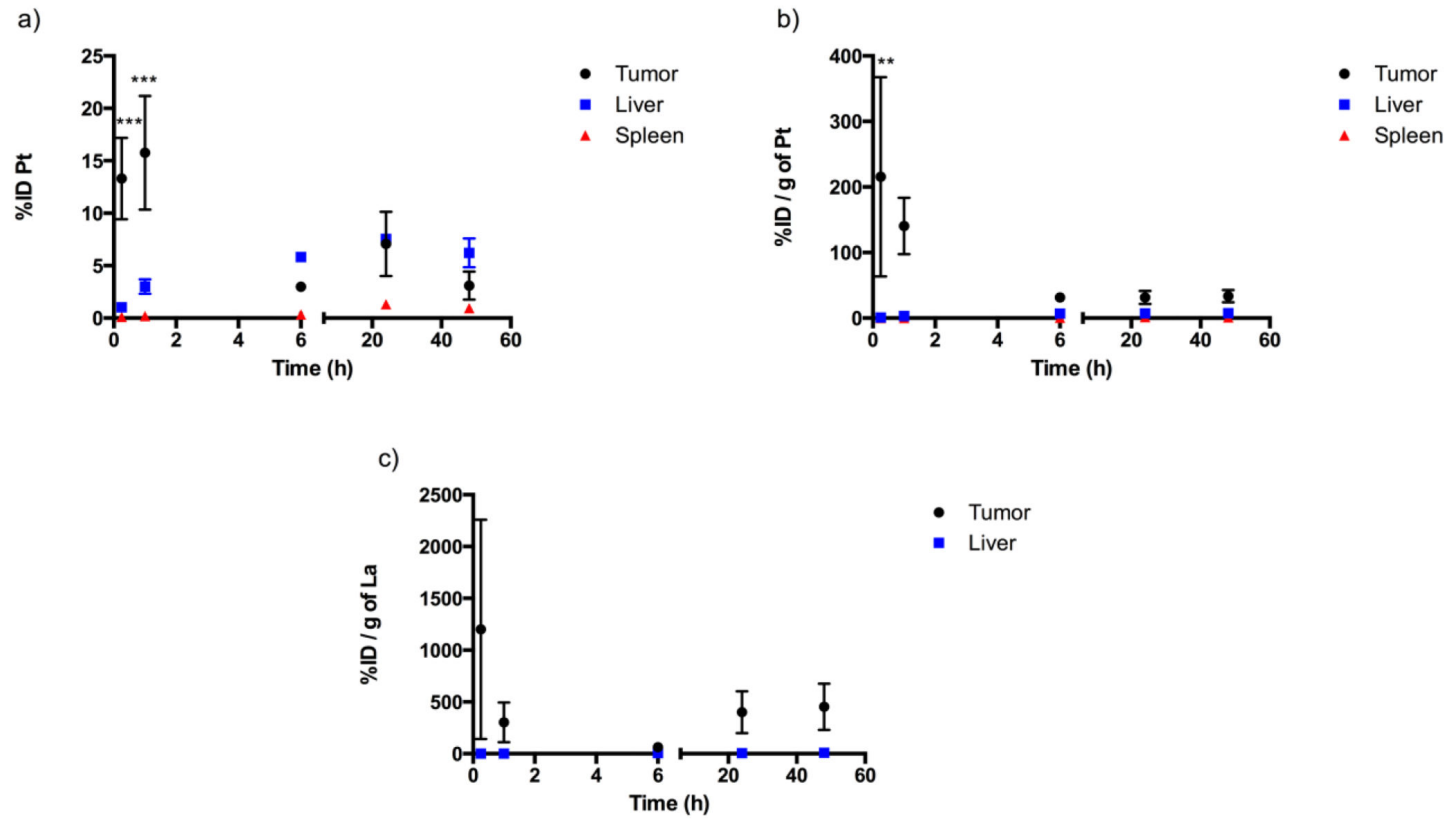
%ID in tumor, liver and spleen were analyzed for both Pt and La. a) %ID of Pt in tumor, liver and spleen; b) Percent injected dose per gram of tissue (%ID/g) of Pt in tumor, liver and spleen; c) %ID/g of La in tumor and liver. (** p<0.01, *** p<0.001, student t-test).

**Figure 7:**
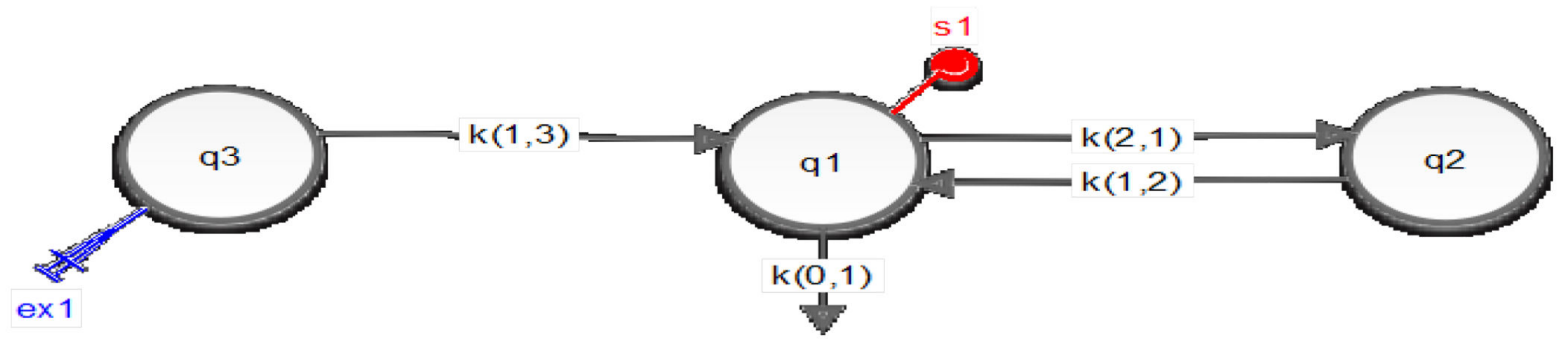
An illustration of a three-compartmental model. Compartments q1, q2 and q3 represent the central plasma, the body tissue and the injection site compartments, respectively. Ex1 and s1 represent the subcutaneous dosing and the blood sampling events, respectively. K(0,1) represents the loss rate constant. K(1,3), k(1,2) and k(2,1) represent the transfer rate constant between compartments

To compare the uptake of drug by the tumor and the drug distribution in highly perfused organs, such as the liver and spleen, we analyzed the data by two different approaches. As shown in panel a) of Figure 6, the percent injected dose (%ID) measured in tumor, liver and spleen were plotted against time. At early time points, such as 0.25 and 1 h, approximately 15% of the injected dose was present in tumor tissue, whereas, less than 5% was present in highly perfused organs including the liver and spleen. This is consistent with a targeted deposition of the drug conjugate, especially as the conjugate is not directly injected into the tumor but is given on the peripheral. Thus a substantial quantity of the conjugate is able to penetrate into the tumor over a short time period. The actual size of the tumor is approximately one tenth of the size of a liver, suggesting an even more pronounced difference if the %ID were normalized per the same amount of the organ tissues (Figure 6b). The localized drug accumulation in tumor tissue may translate into superior efficacy against cancer cells and better penetration to the tumor draining lymphatics. In addition, the targeted drug disposition may spare essential organs, such as liver and kidneys, from harmful side effects of the drug, which directly benefits late stage patients and elderly patients who may already have compromised hepatic or renal function. Another benefit is that as the drug distribution is highly localized in the tumor, a much smaller dose may be administrated, which in turn could reduce the systemic toxicity of the drug.

The pharmacokinetics of the carrier itself was also evaluated and analyzed by plotting the percent injected dose per gram of tissue for La (Figure 6c). As a permanent tag on the polymer backbone, distribution of La indirectly indicated the disposition of the HA carrier. The wide variation in %ID at the first time point may be due to the differences in tumor morphology and allowance of carrier/drug uptake at earlier time points. At later time points, the values are consistent, reflecting penetration of the carriers into the tumors. Furthermore, the %ID/g of La remained at a higher level than that in liver at 24 and 48 h post-dose, suggesting the retention and the continuous uptake of the conjugate into tumor tissue. In comparison, the %ID/g of La was consistently low for all time points for the liver. One mouse was excluded from the modeling due to incomplete blood sampling for the elimination phase, but the AUC was determined for the study timeframe. The V_ss_ was determined to be 0.139 Lkg^−1^. This was significantly lower than the V_ss_ determined in tumor-free rats, which was approximately 3.628 Lkg^−1^ [24]. Besides the interspecies difference between mouse and rat, we believe the conjugate distributed more narrowly in the tumor-bearing mice than the normal rats due to the targeted deposition of the drug in the tumor and the surrounding tissues. In normal rats, though the conjugate demonstrated superior lymphatic penetration, tumor-targeting effect could not be evaluated. In addition, the clearance rate was determined to be 0.0098 Lkg^−1^h^−1^, which was slightly lower than the value determined in normal rats (0.037 Lkg^−1^h^−1^). With regard to the elimination half-lives, the conjugate appeared to be excreted rapidly in tumor-bearing mice; however, it might be attributed to the limited data points available for the drug elimination phase. In our previous study, pharmacokinetic samples were collected until 96 h post-dose, resulting in a more complete drug elimination phase and a superior fit of the data to the model. In the current study, blood samples were collected until 48 h only due to the limited amount of blood volume that can be drawn safely from a mouse without causing discomfort and anemia in a tumor-bearing animal.

## Conclusion

In this work, a novel chemotherapeutic agent, HA-Pt, was directly tagged with traceable amount of La(III) to form a 10-nm nanoconjugate. The *in vitro* release study confirmed that La was strongly bound to HA, and the cytotoxicity of the HA-Pt-La conjugate against cancer cells was comparable as the HA-Pt. The feasibility to use the HA-Pt-La nanoconjugate to achieve the simultaneous chemotherapeutic delivery and HA tracking was demonstrated in the HNSCC tumor-bearing mice.

## Figures and Tables

**Table 1:** Molecular weights and PDIs of NaHA and HA-Pt-La.

Polymer	M_w_ (g/mol)	M_n_ (g/mol)	PDI
NaHA	35,977 ± 5,110	26,601 ± 1,093	1.25 ± 0.11
HA-Pt-La	35,409 ± 4,158	24,484 ± 1,716	1.44 ± 0.14

**Table 2:** Pharmacokinetic parameters of Pt in mice (N=3).

Clearance, L kg^−1^h^−1^	AUC, μg h mL^−1^	t_1/2_ (β), h	V_ss_, L kg^−1^
0.0098±0.0004*	60.3±5.168	0.73±0.69*	0.139±0.038*

* One animal was excluded because there was insufficient data to fit the elimination phase.

## References

[1] Stacker SA, Williams SP, Karnezis T, Shayan R, Fox SB (2014). Lymphangiogenesis and lymphatic vessel remodelling in cancer. Nat Rev Cancer.

[2] Karaman S, Detmar M (2014). Mechanisms of lymphatic metastasis. J Clin Invest.

[3] Franchi A, Gallo O, Massi D, Baroni G, Santucci M (2004). Tumor lymphangiogenesis in head and neck squamous cell carcinoma. Cancer.

[4] Zhang ZA, Helman JI, Li LJ (2010). Lymphangiogenesis, Lymphatic Endothelial Cells and Lymphatic Metastasis in Head and Neck Cancer - A Review of Mechanisms. Int J Oral Sci.

[5] Naor D, Sionov RV, Ish-Shalom D (1997). CD44: structure, function, and association with the malignant process. Adv Cancer Res.

[6] Banerji S, Ni J, Wang SX, Clasper S, Su J (1999). LYVE-1, a new homologue of the CD44 glycoprotein, is a lymph-specific receptor for hyaluronan. J Cell Biol.

[7] Prevo R, Banerji S, Ferguson DJ, Clasper S, Jackson DG (2001). Mouse LYVE-1 is an endocytic receptor for hyaluronan in lymphatic endothelium. J Biol Chem.

[8] Banerji S, Hide BR, James JR, Noble ME, Jackson DG (2010). Distinctive properties of the hyaluronan-binding domain in the lymphatic endothelial receptor Lyve-1 and their implications for receptor function. J Biol Chem.

[9] Jackson DG (2004). Biology of the lymphatic marker LYVE-1 and applications in research into lymphatic trafficking and lymphangiogenesis. APMIS.

[10] Cai S, Xie Y, Davies NM, Cohen MS, Forrest ML (2010). Carrier-based intralymphatic cisplatin chemotherapy for the treatment of metastatic squamous cell carcinoma of the head & neck. Ther Deliv.

[11] Cohen MS, Cai S, Xie Y, Forrest ML (2009). A novel intralymphatic nanocarrier delivery system for cisplatin therapy in breast cancer with improved tumor efficacy and lower systemic toxicity *in vivo*. Am J Surg.

[12] Cai S, Alhowyan AA, Yang Q, Forrest WC, Shnayder Y (2014). Cellular uptake and internalization of hyaluronan-based doxorubicin and cisplatin conjugates. J Drug Target.

[13] Hsieh CM, Huang YW, Sheu MT, Ho HO (2014). Biodistribution profiling of the chemical modified hyaluronic acid derivatives used for oral delivery system. Int J Biol Macromol.

[14] Kim H, Park HT, Tae YM, Kong WH, Sung DK (2013). Bioimaging and pulmonary applications of self-assembled Flt1 peptide-hyaluronic acid conjugate nanoparticles. Biomaterials.

[15] Rosso F, Quagliariello V, Tortora C, Di Lazzaro A, Barbarisi A (2013). Cross-linked hyaluronic acid sub-micron particles: *in vitro* and *in vivo* biodistribution study in cancer xenograft model. J Mater Sci Mater Med.

[16] Ganesh S, Iyer AK, Gattacceca F, Morrissey DV, Amiji MM (2013). *In vivo* biodistribution of siRNA and cisplatin administered using CD44-targeted hyaluronic acid nanoparticles. J Control Release.

[17] Courel MN, Girard N, Chomant J, Liehn JC, Delpech B (1993). 111In-hyaluronectin, a new probe for radiodetection of tumours: biodistribution and imaging in grafted mice. J Nucl Biol Med.

[18] Banzato A, Rondina M, Melendez Alafort L, Zangoni E, Nadali A (2009). Biodistribution imaging of a paclitaxel-hyaluronan bioconjugate. Nucl Med Biol.

[19] Courel MN, Maingonnat C, Bertrand P, Chauzy C, Smadja Joffe F (2004). Biodistribution of injected tritiated hyaluronic acid in mice: a comparison between macromolecules and hyaluronic acid-derived oligosaccharides. In Vivo.

[20] Svanovsky E, Velebny V, Laznickova A, Laznicek M (2008). The effect of molecular weight on the biodistribution of hyaluronic acid radiolabeled with 111In after intravenous administration to rats. Eur J Drug Metab Pharmacokinet.

[21] Mahteme H, Graf W, Larsson BS, Gustafson S (1998). Uptake of hyaluronan in hepatic metastases after blocking of liver endothelial cell receptors. Glycoconj J.

[22] Melendez Alafort L, Riondato M, Nadali A, Banzato A, Camporese D (2006). Bioavailability of 99mTc-Ha-paclitaxel complex [99mTc-ONCOFID-P] in mice using four different administration routes. J Labelled Compd Radiopharmaceut.

[23] Munakata H, Yosizawa Z (1980). Extraction of hyaluronic acid from rabbit skin with lanthanum chloride. Tohoku J Exp Med.

[24] Cai S, Xie Y, Davies NM, Cohen MS, Forrest ML (2010). Pharmacokinetics and disposition of a localized lymphatic polymeric hyaluronan conjugate of cisplatin in rodents. J Pharm Sci.

[25] Moreau J, Guillon E, Pierrard JC, Rimbault J, Port M (2004). Complexing mechanism of the lanthanide cations Eu3+, Gd3+, and Tb3+ with 1,4,7,10-tetrakis(carboxymethyl)-1,4,7,10-tetraazacyclododecane (dota)-characterization of three successive complexing phases: study of the thermodynamic and structural properties of the complexes by potentiometry, luminescence spectroscopy, and EXAFS. Chemistry (Easton).

[26] Hokputsa S, Jumel K, Alexander C, Harding SE (2003). A comparison of molecular mass determination of hyaluronic acid using SEC/MALLS and sedimentation equilibrium. Eur Biophys J.

[27] Xie Y, Bagby TR, Cohen MS, Forrest ML (2009). Drug delivery to the lymphatic system: importance in future cancer diagnosis and therapies. Expert Opin Drug Deliv.

[28] Li WP, Ma DS, Higginbotham C, Hoffman T, Ketring AR (2001). Development of an *in vitro* model for assessing the *in vivo* stability of lanthanide chelates. Nucl Med Biol.

[29] Canales A, Mallagaray A, Berbis MA, Navarro Vazquez A, Dominguez G (2014). Lanthanide-chelating carbohydrate conjugates are useful tools to characterize carbohydrate conformation in solution and sensitive sensors to detect carbohydrate-protein interactions. J Am Chem Soc.

[30] Hascall VEJ (2009). Hyaluronan.

[31] Weigel JA, Raymond RC, Weigel PH (2002). The hyaluronan receptor for endocytosis (HARE) is not CD44 or CD54 (ICAM-1). Biochem Biophys Res Commun.

[32] Fraser JR, Appelgren LE, Laurent T (1983). Tissue uptake of circulating hyaluronic acid. Cell Tissue Res.

